# Supplementary figures and images for: Fundamentals of vaping-associated pulmonary injury leading to severe respiratory distress
Source: Life Sci Alliance. 2021 Nov 22;5(2):e202101246. doi: 10.26508/lsa.202101246 (PMC8616545; doi:10.26508/lsa.202101246)

Full Blots Figure 3C

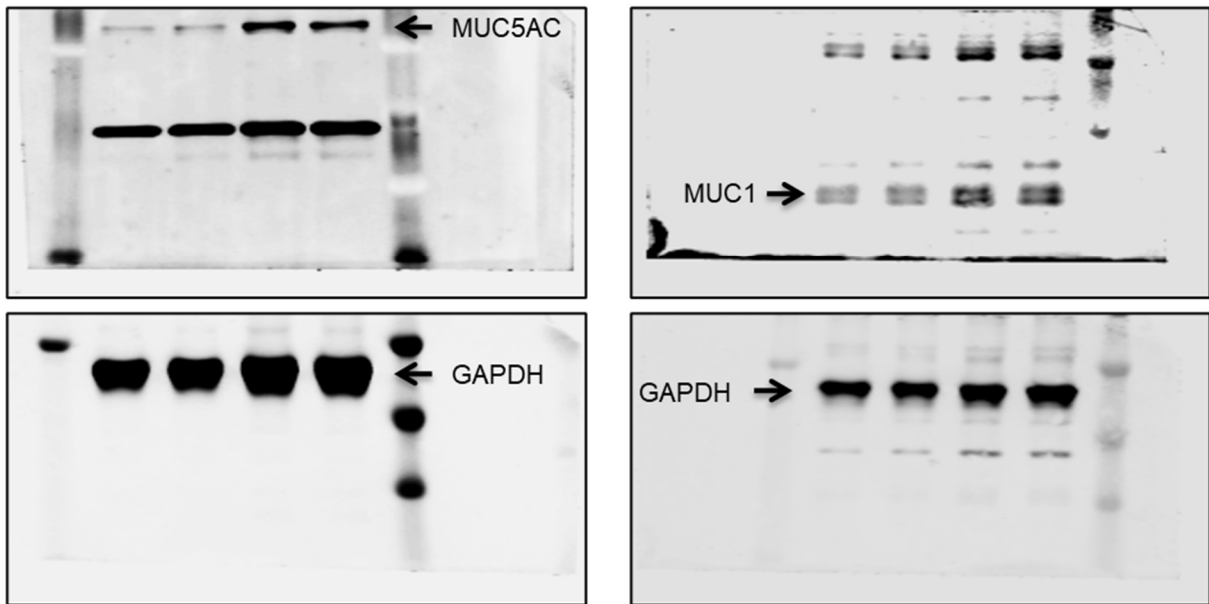

Full Blots Figure 4C

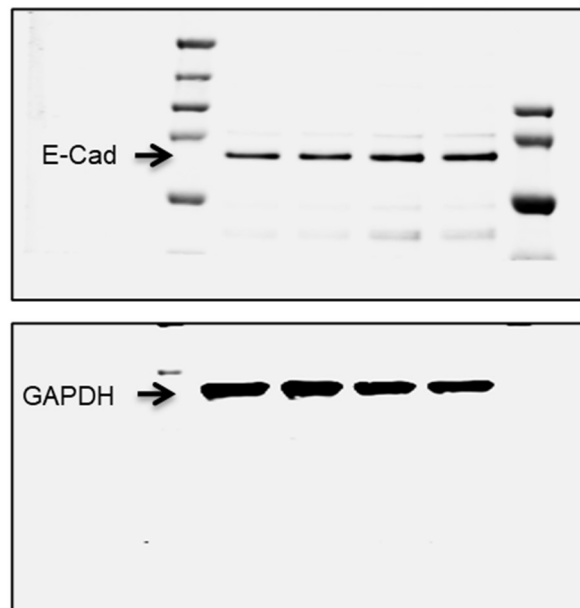

Full Blots Figure 5C

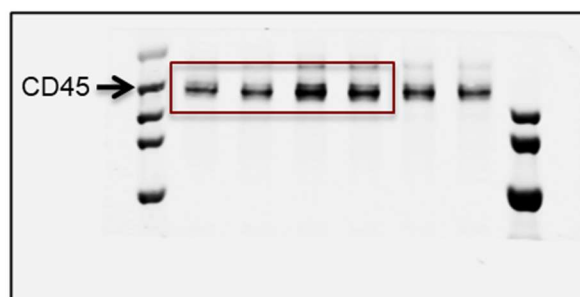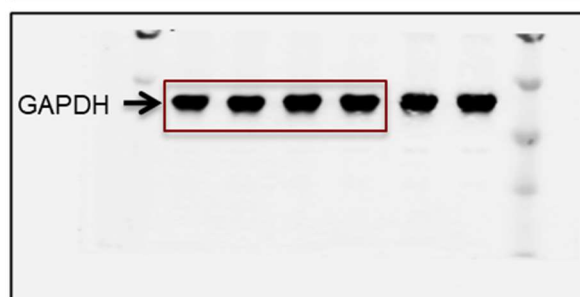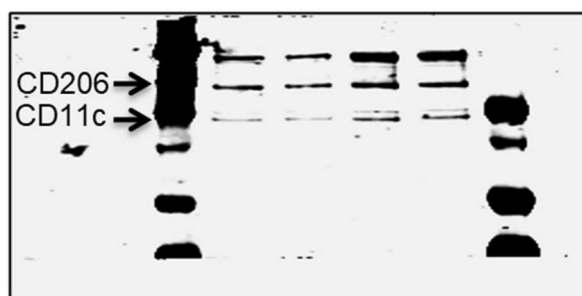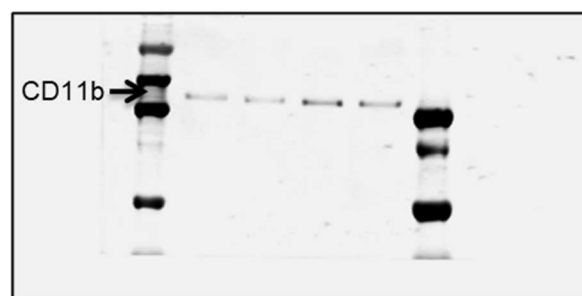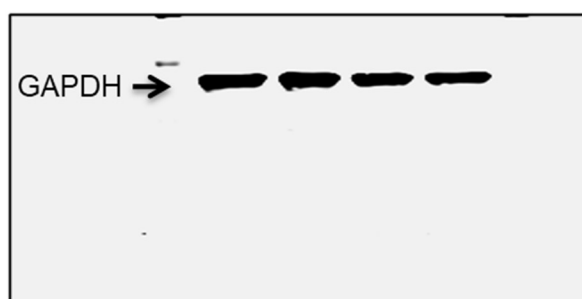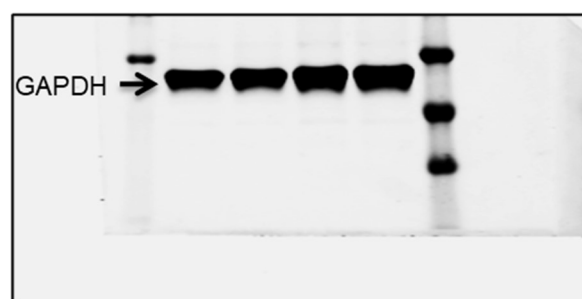

Full Blots Figure 5D

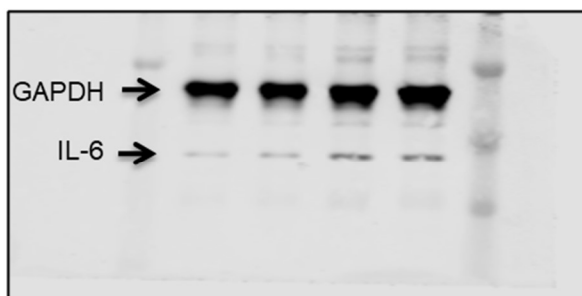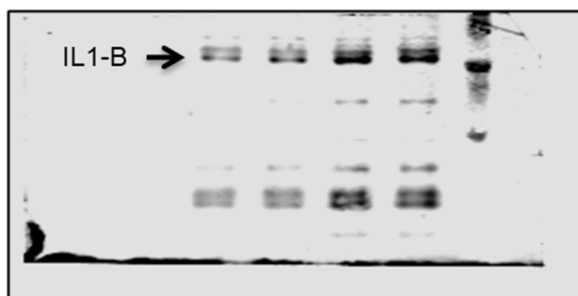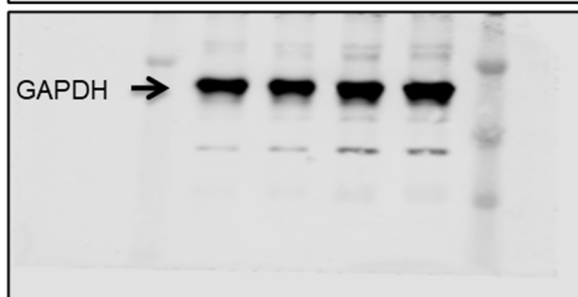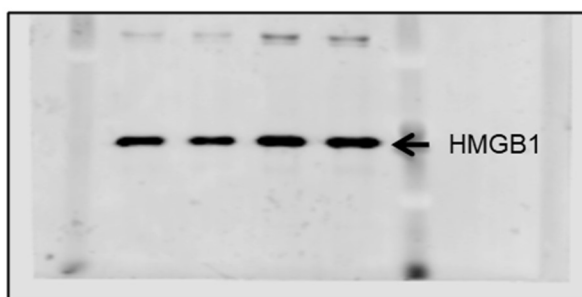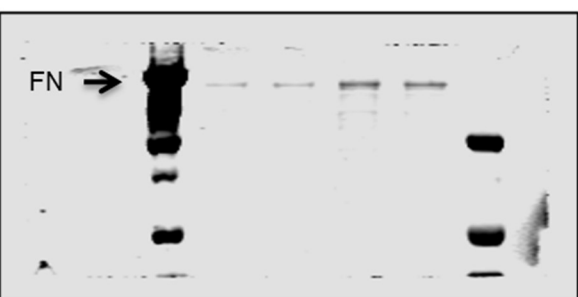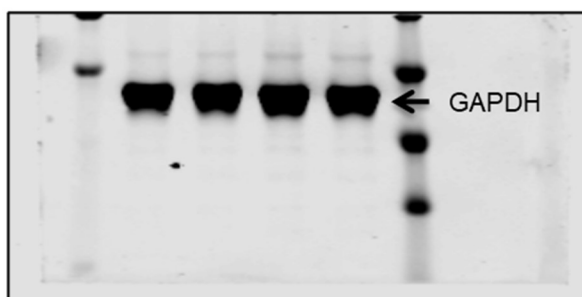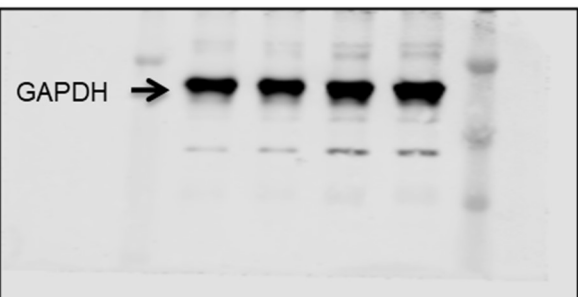

Supplement: Supplementary file 1 [file LSA-2021-01246_SdataF3.F5.pdf]
