# Supplementary material for: Fundamentals of vaping-associated pulmonary injury leading to severe respiratory distress
Source: Life Sci Alliance. 2021 Nov 22;5(2):e202101246. doi: 10.26508/lsa.202101246 (PMC8616545; doi:10.26508/lsa.202101246)
Supplement: Supplementary file 2 [file LSA-2021-01246_TableS1.docx]

Table S1. Antibodies used for immunohistochemistry.

| Marker | Species | Company | Catalog # | Dilution |
| --- | --- | --- | --- | --- |
| ECAD | Goat | R&D Systems | AF748 | 1:100 |
| Vimentin | Chicken | Invitrogen | PA1-10003 | 1:400 |
| CD11C | Rabbit | Cell Signaling Tech | 97585S | 1:200 |
| RAGE | Goat | R&D Systems | AF1145 | 1:100 |
| KRT5 | Chicken | Biolegend | 905901 | 1:200 |
| AQ5 | Rabbit | Abcam | Ab78486 | 1:400 |
| CD9 | Rabbit | Abcam | Ab92726 | 1:100 |
| Alpha Tubulin | Rat | Biorad | 1703 | 1:400 |
| Muc5ac | Rabbit | US Biotech | 1364248 | 1:100 |
| Desmin | Rabbit | Abcam | Ab15200 | 1:200 |
