# Supplementary material for: Fundamentals of vaping-associated pulmonary injury leading to severe respiratory distress
Source: Life Sci Alliance. 2021 Nov 22;5(2):e202101246. doi: 10.26508/lsa.202101246 (PMC8616545; doi:10.26508/lsa.202101246)
Supplement: Supplementary file 3 [file LSA-2021-01246_TableS2.docx]

Table S2. Echocardiographic measurements shown by experimental group and gender. Values represent mean ± standard deviation.

|  | No Vape Female | No Vape Male | Vape Female | Vape Male |
| --- | --- | --- | --- | --- |
| EF | 74.82± 0.464655 | 74.99±2.531607 | 64.95±3.74678 | 59.43±2.98888 |
| FS | 42.24±0.476157 | 42.66±2.090859 | 34.4±2.743135 | 30.66±1.985322 |
| LV_Mass | 81.53±18.11191 | 89.68±13.87718 | 80.95±4.805302 | 88.3±11.93415 |
| LV_Vold | 35.42±6.053918 | 42.03±7.525448 | 36.39±4.106111 | 44.07±6.537644 |
| LV_Vols | 8.916±1.502419 | 10.612±2.718028 | 12.75±1.988373 | 17.92±3.238416 |
| LVIDd | 3.007±0.212416 | 3.225±0.239866 | 3.045±0.139681 | 3.291±0.207086 |
| LVIDs | 1.736±0.115514 | 1.852±0.185913 | 1.997±0.119995 | 2.283±0.167831 |
| IVSd | 0.9405±0.08526 | 0.957±0.042974 | 0.8723±0.048719 | 0.9123±0.022281 |
| IVSs | 1.355±0.098609 | 1.382±0.093474 | 1.3±0.060127 | 1.265±0.108597 |
| LVPWd | 0.75±0.171771 | 0.726±0.035867 | 0.8028±0.043058 | 0.718±0.111153 |
| LVPWs | 0.8085±0.184679 | 0.7938±0.072507 | 0.8578±0.063111 | 0.7594±0.064659 |
