# Supplementary material for: Fundamentals of vaping-associated pulmonary injury leading to severe respiratory distress
Source: Life Sci Alliance. 2021 Nov 22;5(2):e202101246. doi: 10.26508/lsa.202101246 (PMC8616545; doi:10.26508/lsa.202101246)
Supplement: Supplementary file 4 [file LSA-2021-01246_TableS3.docx]

Table S3. Parameters for cardiomyocyte length measurements.

| Male Vape | | Female Vape | | Male No Vape | | Female No Vape | |
| --- | --- | --- | --- | --- | --- | --- | --- |
| Sample # | Measurements | Sample # | Measurements | Sample # | Measurements | Sample # | Measurements |
| 21053 | 25 | 21075 | 23 | 21061 | 29 | 21071 | 24 |
| 21099 | 38 | 21077 | 28 | 21042 | 19 | 21041 | 20 |
| 21044 | 43 | 21043 | 34 | 21113 | 35 | 21059 | 28 |
| 21065 | 27 | 21089 | 31 |  |  |  |  |
| Total Measurements: 133 | | Total Measurements: 116 | | Total Measurements: 83 | | Total Measurements: 72 | |
| Mean: 62.51 | | Mean: 60.93 | | Mean: 56.92 | | Mean: 53.76 | |
| % Increase: 8.94 | | % Increase: 11.77 | |  | | | |
