# Supplementary material for: Fundamentals of vaping-associated pulmonary injury leading to severe respiratory distress
Source: Life Sci Alliance. 2021 Nov 22;5(2):e202101246. doi: 10.26508/lsa.202101246 (PMC8616545; doi:10.26508/lsa.202101246)
Supplement: Supplementary file 5 [file LSA-2021-01246_TableS4.docx]

Table S4. Antibodies used for immunoblotting.

| Marker | Species | Company | Catalog # | Dilution |
| --- | --- | --- | --- | --- |
| CD11b | Rabbit | Abcam | Ab133357 | 1:500 |
| CD11c | Rabbit | Cell Signaling | 97585s | 1:500 |
| CD206 | Rabbit | Abcam | Ab64693 | 1:500 |
| CD45 | Goat | R&D | AF114 | 1:500 |
| E-Cadherin | Rabbit | R&D | AF748 | 1:500 |
| Fibronectin | Rabbit | Sigma | F3648 | 1:250 |
| GAPDH | Goat | Sicgen | AB0067 | 1:3000 |
| HMGB1 | Rabbit | Abcam | Ab18256 | 1:250 |
| IL1-β | Rabbit | Invitrogen | P420B | 1:50 |
| IL-6 | Mouse | Sino Biological | 10395-MM19 | 1:250 |
| MUC1 | Rabbit | Abcam | Ab109185 | 1:250 |
| MUC5AC | Rabbit | My Biosource | MBS2028179 | 1:200 |
